# ‘Hangry’ *Drosophila*: food deprivation increases male aggression

**DOI:** 10.1016/j.anbehav.2021.05.001

**Published:** 2021-07

**Authors:** Danielle Edmunds, Stuart Wigby, Jennifer C. Perry

**Affiliations:** a Department of Zoology, University of Oxford, U.K.; b Department of Evolution, Ecology, and Behaviour, Institute of Infection, Veterinary & Ecological Sciences, University of Liverpool, U.K.; c School of Biological Sciences, University of East Anglia, U.K.

**Keywords:** aggression, contest theory, diet, *Drosophila melanogaster*, nutrition

## Abstract

Aggressive interactions are costly, such that individuals should display modified aggression in response to environmental stress. Many organisms experience frequent periods of food deprivation, which can influence an individual's capacity and motivation to engage in aggression. However, because food deprivation can simultaneously decrease an individual's resource-holding potential and increase its valuation of food resources, its net impact on aggression is unclear. Here, we tested the influence of increasingly prolonged periods of adult food deprivation on intermale aggression in pairs of fruit flies, *Drosophila melanogaster*. We found that males displayed increased aggression following periods of food deprivation longer than a day. Increased aggression in food-deprived flies occurred despite their reduced mass. This result is probably explained by an increased attraction to food resources, as food deprivation increased male occupancy of central food patches, and food patch occupancy was positively associated with aggression. Our findings demonstrate that aggressive strategies in male *D. melanogaster* are influenced by nutritional experience, highlighting the need to consider past nutritional stresses to understand variation in aggression.

## Highlights

• Limited food can lower resource-holding potential and raise resource valuation.
• The net impact of food deprivation on aggression is unclear.
• We found that adult food deprivation increases aggression in male fruit flies.
• Deprived males occupied food for longer, which related to higher aggression.
• Dietary stress increases aggression, suggesting flies get ‘hangry’.

Aggressive contests occur throughout the animal kingdom and involve a wide range of agonistic behaviours, from noncontact threat displays to escalated physical fights (Briffa, Sneddon, & Wilson, 2015; Briffa & Sneddon, 2007). Aggressive contests typically occur over resources, such as food, territories and mates, that are critical for reproduction (Clutton-Brock & Albon, 1979; Stockley & Campbell, 2013; Bergman, Olofsson, & Wiklund, 2010; Hoffmann & Cacoyianni, 1990; Zwarts, Versteven, & Callaerts, 2012). Aggression is costly, carrying the risk of physical damage and predation, along with time and energy expenditure (Briffa & Sneddon, 2007; Haley, 1994; Neat, Taylor, & Huntingford, 1998). Contest theory suggests that aggression should be expressed according to an individual's relative fighting ability (resource-holding potential) and perceived value of the contested resource (resource valuation; Briffa & Sneddon, 2007; Enquist & Leimar, 1987).

A key factor shaping resource-holding potential and resource valuation is an individual's nutritional experience. Access to nutritional resources varies, and animals often experience periods of food deprivation (Wang, Hung, & Randall, 2006). Food deprivation can have a long-term impact on internal state, determining an individual's ability to invest in life history traits (Rowe & Houle, 1996; Scharf, 2016) and affecting size, physiology and behaviour (Han & Dingemanse, 2015, 2017; Harrison, Godin, & Bertram, 2017; Lihoreau et al., 2015). Furthermore, food limitation can signal information about the physical and social environment, such as the characteristics of potential mates (Gibson & Uetz, 2012; Zikovitz & Agrawal, 2013) and rivals (Delisle & Hardy, 1997; Engels & Sauer, 2007; Fricke, Bretman, & Chapman, 2008) and the future environment for potential offspring (Kotiaho, Simmons, & Tomkins, 2001; Tudor, Promislow, & Arbuthnott, 2018; Zirbel & Alto, 2018).

However, because food deprivation can simultaneously decrease resource-holding potential and increase the resource valuation, the expected net impact on aggression is often unclear (Stocker & Huber, 2001). Food deprivation can reduce resource-holding potential by compromising traits that determine fighting ability, such as body size, weapon-like appendages and energy reserves (Briffa & Sneddon, 2007; Plaistow & Siva-Jothy, 1996; Baker et al., 2003; Poças, Crosbie, & Mirth, 2020; Marden & Waage, 1990; Plaistow & Siva-Jothy, 1996, 1996). On the other hand, food deprivation might increase motivation to engage in escalated and persistent aggression to gain food resources (Elias, Botero, Andrade, Mason, & Kasumovic, 2010; Enquist & Leimar, 1987; McNamara & Houston, 1989; Stocker & Huber, 2001). Results from previous studies are mixed, with food deprivation sometimes increasing (e.g. Nosil, 2002), decreasing (e.g. Griffiths, 1992) or having no effect on aggression (Trumbo, 2012; Weiß, Kramer, Holländer, & Meunier, 2014). Furthermore, the balance between the opposing influences of food deprivation on fighting capacity and motivation might vary with the severity of food deprivation (Scharf, 2016). Because food deprivation in nature can span brief to prolonged periods, it is important to understand how aggression changes along a continuous gradient of food deprivation.

We tested how adult food deprivation influences male aggression and food patch occupancy in the fruit fly, *Drosophila melanogaster*. Aggression is a key social behaviour for both male and female *D. melanogaster* (Nilson, Chan, Huber, & Kravitz, 2004). In males, aggression has an important function in mate acquisition (Hoffmann, 1987a, 1987b; Kravitz & Fernandez, 2015). Contests often occur over food sources, which represent not only nutrition, but also high-value mating sites (Hoffmann & Cacoyianni, 1990; Markow, 1988). Because *D. melanogaster* consume decaying fruits, which are seasonally and spatially variable, nutritional quality and quantity vary in natural settings (Chng, Hietakangas, & Lemaitre, 2017; Markow, 1988). Both nutrient quantity and quality might influence aggression; here, we focused on quantity because periods of food deprivation are common in natural insect populations (Scharf, 2016). Adult nutrition affects male postcopulatory reproductive success (Fricke et al., 2008), but nutritional effects on precopulatory interactions via intermale aggression are unknown. We hypothesized that exposure to food deprivation might decrease aggression by reducing male resource-holding potential, or might increase aggression by increasing resource valuation and motivation, and that these alternative outcomes might depend on the duration of food deprivation.

## Methods

### Experimental Flies

**Figure A1:**
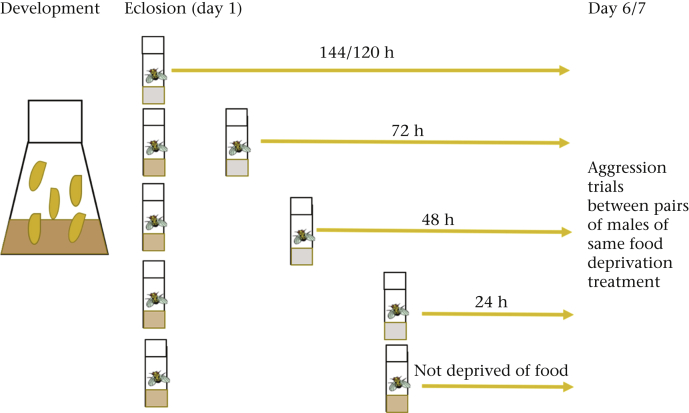
A schematic representation of the food deprivation treatment. Flies developed on standard food medium in bottles at a density of 200/bottle. At eclosion, flies assigned to the ‘food deprivation from eclosion’ treatment were placed singly in food deprivation vials containing only agar (grey medium). Flies of all other treatments were placed singly in standard food medium vials at eclosion (pink medium). Flies were either transferred to food deprivation vials 72, 48 or 24 h before behavioural trials, or, if assigned to the ‘no food deprivation’ treatment, were transferred to food vials as a handling control 24 h before behavioural trials. Behavioural trials were carried out when flies were 6–7 days old. Pairs of flies of the same treatment were placed in vials containing agar and a central patch of standard food medium and yeast.

Flies were derived from an outbred Dahomey stock population (Carazo, Perry, Johnson, Pizzari, & Wigby, 2015). Fly husbandry and experiments were carried out at 25 ^o^C on a 12:12 h light:dark cycle. Experimental flies were reared at a density of 200/bottle. We collected virgin males within 6 h of eclosion using ice anaesthesia. We conducted the experiment in three blocks. We randomly assigned males to one of five treatments: food deprivation from eclosion (120–144 h; included in blocks 2 and 3 only; total *N* = 24) or for 72 h (*N* = 58), 48 h (*N* = 59) or 24 h (*N* = 62), or no food deprivation (*N* = 62; Fig. A1). No flies died during the experiment.

We placed males that were assigned to food deprivation from eclosion singly in vials lacking nutritional substances but containing agar for moisture. We placed all other males singly in vials containing standard food medium (Table A1) and transferred them to agar vials at the assigned number of hours before trials. As a handling control, we transferred males assigned to ‘no food deprivation’ to new food vials 24 h before trials.

### Behavioural Trials

We transferred pairs of flies from each treatment into vials containing agar with a central 0.2 cm diameter patch of food medium combined with yeast paste, with 1.5 cm between the agar and cotton bung for flies to interact. Flies were 6 or 7 days old at the time of trials. We allowed flies 10 min acclimatization before trials. During trials, an observer blind to treatment scanned each vial a total of 16 (block 1) or 32 (block 2–3) times. Each scan lasted 3 s. In each scan, the observer recorded the number of lunges and tussles and the number of flies chasing, fencing and occupying the food patch in each scan (Table A2; Andrews, 2016; Dow & von Schilcher, 1975; Nilsen et al., 2004). We carried out trials for 5 h from lights-on. We froze males at -20 ^o^C immediately after trials and weighed each pair before and after drying for 48 h at 60 ^o^C.

### Statistical Analysis

We performed analyses in R version 3.6.2 (The R Foundation for Statistical Computing, Vienna, Austria, http://www.r-project.org). We converted spot check data into both bouts/min (‘behaviour rates’) and binary responses (when data were zero-inflated) to describe whether the behaviour occurred.

We assessed the effects of food deprivation duration on the total aggression rate (lungeing, fencing, chasing and tussling) in a linear model. We found an effect of the food deprivation treatment on total aggression, so we next explored the effect of food deprivation duration on lungeing, fencing and chasing in separate models. Because these behaviours were zero-inflated, we modelled the probability that each aggressive behaviour was performed during behavioural observations using binomial general linear models for binary data. We excluded tussling from individual analysis, as it was only observed in two of 265 pairs. Chi-square analysis revealed that the probability of lungeing, chasing and fencing were not independent, but co-occurred within individuals more frequently than expected by chance (lungeing–chasing: χ^2^ = 129.8, *P* < 0.0001; lungeing–fencing: χ^2^ = 20.9, *P* < 0.0001; chasing–fencing: χ^2^ = 31.3, *P* < 0.0001). We therefore applied a Bonferroni correction to adjust the threshold for statistical significance for these three models (α/3 = 0.0167), to reduce the scope for type I errors. We analysed the effects of food deprivation duration on mass, measured as the mean mass of the two males per vial, and the relationship between mass and total aggression, in separate linear models. We conducted sequential sum of squares analysis (type I ANOVA; Whitlock & Schluter, 2009) to test for effects of mass after food deprivation had been accounted for.

We analysed the effects of food deprivation duration on food patch occupancy rates in a linear model. We tested the relationship between aggression rates and food patch occupancy using Spearman rank correlation because the data were not normally distributed (Shapiro–Wilk: aggression: *W* = 0.74, *P* < 0.0001; food patch occupancy: *W* = 0.88, *P* < 0.0001). We conducted sequential sum of squares analysis to test for effects of food patch occupancy on aggression after food deprivation had been accounted for.

To test for the potential influence of outliers, we refitted models using winsorized data, and, as this had minimal impact on the results, we report all statistics for nonwinsorized data (Carazo et al., 2015). We included block as a fixed factor in all models and used post hoc Tukey adjusted pairwise comparisons (emmeans package, Lenth, 2020) to explore the results.

### Ethical Note

We used laboratory-maintained insects for which no licences, permits or ethical approval were required. This research was conducted in accordance with ASAB/ABS Guidelines for the Use of Animals in Research.

## Results

### Food Deprivation Increased Aggression

**Figure 1:**
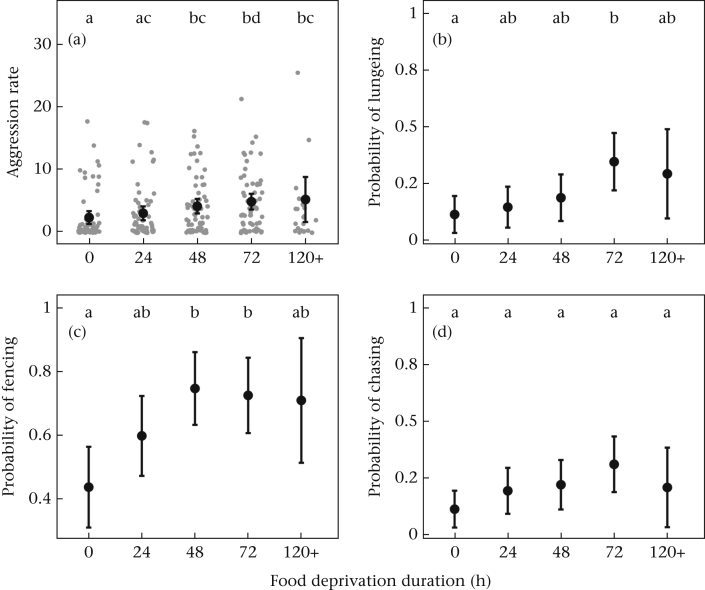
Relationships between food deprivation and (a) total aggression rate (aggressive bouts/min), and the probability of (b) lungeing, (c) fencing and (d) chasing (results from binomial models). Of 265 pairs, 54 performed lungeing, 167 performed fencing and 55 performed chasing. Black points show means with 95% confidence intervals from model output, estimated based on the number of trials. (a) Grey points show the raw data. Letters denote significant differences between groups by post hoc Tukey adjusted pairwise comparisons.

Food deprivation influenced aggression (*F*_4,258_ = 6.4, *P* < 0.0001; block: *F*_2,258_ = 7.8, *P* < 0.001; Fig. 1). We observed a trend of increasing aggression with longer food deprivation, with significant differences between males with full access to food and those experiencing ≥48 h food deprivation (see Fig. 1a for post hoc results). Likewise, the likelihood of lungeing and fencing was influenced by food deprivation duration (lungeing: χ^2^_4,258_ = 13.3, *P* = 0.010; block: *F*_2,258_ = 1.8, *P* = 0.405; fencing: χ^2^_4,258_ = 17.5, *P* = 0.002; block: *F*_2,258_ = 3.4, *P* = 0.183; Fig. 1b and c), although chasing was unaffected (χ^2^_4,258_ = 7.4, *P* = 0.115; block: χ^2^_2,258_ = 0.2, *P* = 0.925; Fig. 1d).

**Figure 2:**
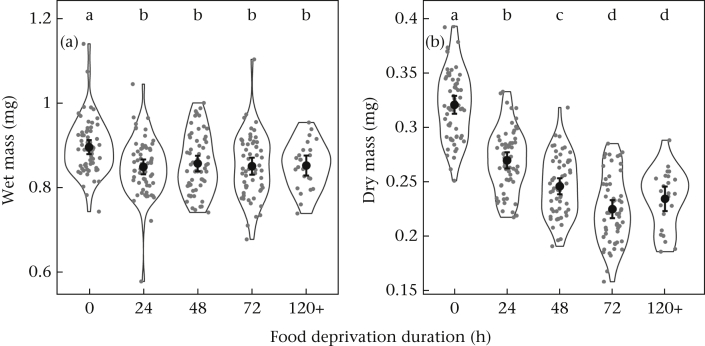
The effect of food deprivation duration on (a) wet and (b) dry mass. Black points show means with 95% confidence intervals, grey points show the raw data and ‘violin’ shapes show the shape of the distribution. Letters denote significant differences between groups by post hoc tests.

**Figure A2:**
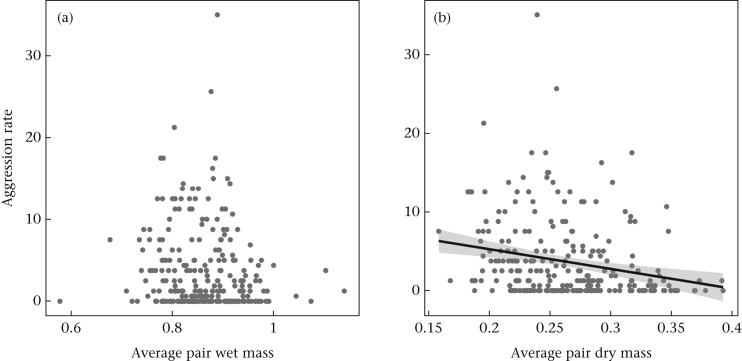
The relationship between the mean (a) wet mass and (b) dry mass of a pair of males and the observed rate of aggression (bouts/min).

Food deprivation might influence body mass, which in turn might influence behaviour. We therefore assessed the effect of food deprivation on wet and dry mass because both might influence aggression (e.g. success in contests might relate to total wet mass or to muscle mass). We observed a reduction in wet mass after 24 h of food deprivation (*F*_4,257_ = 7.9, *P* < 0.0001; block: *F*_2,257_ = 23.5, *P* < 0.0001), with no further reduction with longer food deprivation, whereas dry mass decreased further after 48 and 72 h (*F*_4,252_ = 189.9, *P* < 0.0001; *F*_2,252_ = 105.9, *P* < 0.0001; Fig. 2). Total aggression was negatively related to mean dry mass for a pair (*F*_1,255_ = 14.4, *P* = 0.0002; *F*_2,255_ = 2.0, *P* = 0.131), but we detected no relationship with wet mass (*F*_1,260_ = 0.7, *P* = 0.397; block: *F*_2,260_ = 3.9, *P* = 0.021; Fig. A2). However, sequential sum of squares analysis revealed that the negative relationship between dry mass and aggression was no longer detectable after accounting for food deprivation: dry mass was positively related to the variation in aggression that was not explained by food deprivation (*F*_1,251_ = 4.0, *P* = 0.046, slope = 3.01 ± 3.56).

### Food Deprivation Increased Food Patch Occupancy

**Figure 3:**
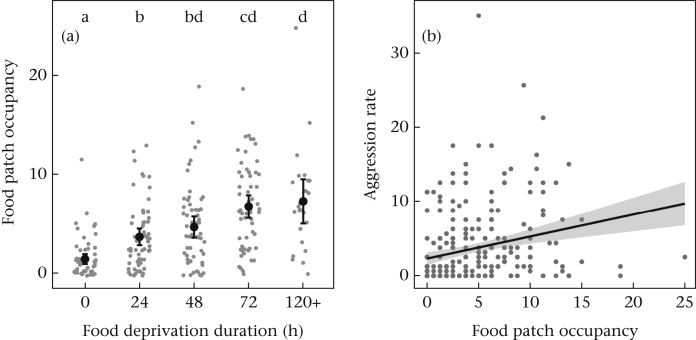
The relationships between (a) food deprivation and food patch occupancy (bouts/min) and (b) food patch occupancy and total aggression rate (both in bouts/min). In (a), black points show means with 95% confidence intervals and grey points show the raw data, and in (b), the line is plotted from the correlation analysis, with grey areas representing 95% confidence intervals. Letters denote significant differences between groups by post hoc tests.

Food deprivation influenced food patch occupancy (*F*_4,258_ = 23.4, *P* < 0.0001; block: *F*_2,258_ = 5.6, *P* = 0.004). Males experiencing any food deprivation spent more time on the food patch than those with full access to food, with further increases in food patch occupancy with prolonged food deprivation (Fig. 3a). Food patch occupancy was positively correlated with aggression (*ρ* = 0.38, *P* < 0.0001; Fig. 3b). Sequential sum of squares analysis revealed that the positive relationship between food occupancy and aggression remained after accounting for the influence of food deprivation, with food occupancy positively correlating with the variation in aggression that was not explained by food deprivation (*F*_1,257_ = 12.9, *P* = 0.0004).

## Discussion

Periods of food deprivation are common in many animals, and so plastic behavioural strategies to mitigate the negative consequences of nutritional stress are also common (Monaghan, 2008). We found that prolonged food deprivation increases both male aggression and food patch occupancy in *D. melanogaster*, and that aggression and food patch occupancy behaviours were positively correlated. Increased aggression following food deprivation occurred above and beyond effects of the reduced body mass resulting from food deprivation. These findings support the hypothesis that prolonged food deprivation increases aggression through increasing resource valuation and motivation and are consistent with elevated aggression following food deprivation in other organisms (reviewed in Scharf, 2016). Our results demonstrate that males modify their aggression in response to nutritional experience, consistent with the predictions from the hypothesis of increased resource valuation and motivation (Bretman, Fricke, Westmancoat, & Chapman, 2016; Fusco & Minelli, 2010). In popular parlance, food-deprived male fruit flies get ‘hangry’.

### Increased Resource Valuation

Increased aggression by food-deprived males could be explained by increased valuation of nutritional resources, and hence increased motivation to access those resources. Consistent with this hypothesis, we observed increased food patch occupancy with extended food deprivation, and this was correlated with increased aggression. Attraction to food resources is influenced by nutritional status in many organisms, including humans (Aimé et al., 2007; Farhadian, Suárez-Fariñas, Cho, Pellegrino, & Vosshall, 2012; Uher, Treasure, Heining, Brammer, & Campbell, 2006). Although male *D. melanogaster* gain most of their lifetime nutrition in larval development (Edgar, 2006), adult feeding is necessary to develop internal reproductive structures and to maximize mating success (Baker et al., 2003; Fricke et al., 2008). Sexual maturation occurs in the days following eclosion (Eastwood & Burnet, 1977; Markow & O'Grady, 2008), and if food deprivation slows this process, then food-deprived males might increase their food valuation to support completion of development.

Our results suggest that at least part of the increase in aggression following food deprivation might result from greater male–male proximity with increased occupation of the food patch. Increased food patch occupancy might have resulted from the heightened sensitivity to food odours after food deprivation in *D. melanogaster* (Edgecomb, Harth, & Schneiderman, 1994; Farhadian et al., 2012). Greater food patch occupation might increase intermale aggression in *D. melanogaster* via the action of Gr5a+ gustatory receptor neurons and octopamine signalling (Andrews, 2016; Lim, Eyjólfsdóttir, Shin, Perona, & Anderson, 2014). Thus, the increased aggression displayed by food-deprived males is coupled with increased occupation of food patches, triggered by an increased attraction to food odours, allowing increased access to food following deprivation.

### Increased Motivation to Access Mating Sites

If prolonged periods of food deprivation signal a reduced likelihood of survival (Good & Tatar, 2001; Tigreros, 2013), then males should invest more in immediate reproductive effort (i.e. terminal investment, Clutton-Brock, 1984; Krams et al., 2015; Moatt, Nakagawa, Lagisz, & Walling, 2016). Aggression in male *D. melanogaster* can occur over access to mates (Hoffmann, 1987b; Kravitz & Fernandez, 2015; Nilsen et al., 2004) and food patches are important for access to females, which are attracted to nutritionally rich oviposition sites (Hoffmann & Cacoyianni, 1990; Lim et al., 2014; Markow, 1988). Thus, increased aggression by food-deprived males might be a strategy to maximize short-term reproductive output in environments where survival is uncertain. Further investigation into how aggression influences the reproductive output of food-deprived males could shed light on this hypothesis.

### No Strong Support for Decreased Resource-Holding Potential

Our findings provide no strong evidence that adult food deprivation decreases resource-holding potential. Body size is a common correlate of resource-holding potential (Asahina, 2017; Kemp & Alcock, 2003; Stockermans & Hardy, 2013), and larger mass can increase aggressive initiation, escalation and success in *D. melanogaster* (Asahina et al., 2014; Bath, Morimoto, & Wigby, 2018; Hoffmann, 1987b; Hoyer et al., 2008) and other species (DiMarco & Hanlon, 1997; Kelly, 2008; McCann, 1981; Schuett, 1997). We found that adult food deprivation decreased body mass, with reduced dry mass suggesting the depletion of fat or structural protein (Kristensen, Overgaard, Loeschcke, & Mayntz, 2011; Robinson, Zwaan, & Partridge, 2000). However, these lighter, food-deprived males displayed elevated aggression. Thus, increased resource valuation caused by dietary restriction might override any reduction in resource-holding potential (e.g. Nosil, 2002). Alternatively, increased aggression in food-deprived males might result from a ‘desperado’ effect, in which individuals of poor condition engage in fights even when likely to lose, because they cannot gain fitness benefits by not engaging at all (Elias et al., 2010; Grafen, 1987).

### A Monotonic Relationship Between Food Deprivation and Aggression

We speculated that the direction of the relationship between food deprivation and aggression might depend on the severity of food deprivation. Food deprivation can cause a reallocation of resources from reproduction to survival, delaying reproduction until conditions improve (Shanley & Kirkwood, 2000), and brief food deprivation might result in individuals decreasing aggression to conserve resources. However, severe food deprivation that reduces survival might trigger a terminal investment in reproduction (Shanley & Kirkwood, 2000), increasing aggressive motivation to attain resources before death. Conversely, starvation might render individuals too weak to fight, while brief food deprivation might increase aggressive motivation before decreased resource-holding potential occurs. These processes would generate a nonlinear relationship between food stress and aggression. Similar nonlinear responses have been reported for male postcopulatory success: male *D. melanogaster* siring success is maximized under intermediate levels of adult nutrition (Fricke et al., 2008), and aggression peaks at intermediate food patch size (Lim et al., 2014). Our results did not reveal a U-shaped or inverse U-shaped relationship between food deprivation and aggression, but a continuous decrease in aggression as food deprivation duration extended beyond 24 h. This suggests that increased resource valuation might be the strongest consequence of adult food deprivation, resulting in increased aggressive motivation despite any reduction in fighting capacity. Alternatively, our food deprivation treatments might not have been severe enough to capture a switch-point driven by terminal investment; indeed, no experimental males died following our treatments, showing a similar survival duration under food deprivation conditions as previously reported in *D. melanogaster* of a Dahomey background (Bjedov et al., 2010; Broughton et al., 2005). This, combined with our observation that food deprivation longer than 24 h was necessary to decrease aggression, suggests that 24 h without food is well tolerated and that up to 120 h without food does not compromise survival in adult male *D. melanogaster*.

Our findings that adult food deprivation increases aggression and food patch occupancy in male *D. melanogaster* demonstrate that behavioural strategies critically depend on their nutritional experience, even in adult insects with low food requirements. The observed behavioural responses reflect patterns of increased aggression associated with starvation in a wider range of organisms (e.g. Arnott & Elwood, 2008; Nosil, 2002; Scharf, 2016). These results highlight the need to consider the environmental stresses experienced in the recent past to understand adaptive variation in behaviour.

## Author Contributions

D.E., S.W. and J.C.P. conceived the ideas and designed the methodology; D.E. collected the data; D.E., S.W. and J.C.P. analysed the data; D.E. drafted the initial manuscript and all authors contributed to the final manuscript.

## Data Availability

Data are available from ORA, DOI: 10.5287/bodleian:xrO2DD55e.

## Figures and Tables

**Table A1:** Standard food medium (values per litre of medium)

Ingredients	Amount/litre
Agar (g)	6.923
Maize (g)	69.231
Soya (g)	8.308
Yeast (g)	14.077
Malt (g)	69.231
Molasses (ml)	23.077
Nipagin (ml)	30.000
Acid mix (propionic acid + orthophosphoric acid, ml)	5.385

**Table A2:** Description of male aggressive behaviours

Aggressive behaviours	Description
Lungeing	The male rises up on hindlegs and rapidly thrusts his upper body at his opponent
Chasing	One male rapidly pursues his opponent, remaining in close proximity. Contact and even aggressive lungeing may occur during chasing
Tussling	Both opponents rise up on hindlegs and become interlocked in a prolonged aggressive struggle
Fencing	A male uses his forelimbs to bat his opponent. This includes front-on and side-on action with any of the two front or middle legs, and can be performed by one or both opponents, alone or in combination with other behaviours

From Andrews, 2016; Dow & von Schilcher, 1974; Nilsen et al., 2004.
